# Supplementary material for: The Role of Gut Microbiome in Psoriasis: Oral Administration of Staphylococcus aureus and Streptococcus danieliae Exacerbates Skin Inflammation of Imiquimod-Induced Psoriasis-Like Dermatitis
Source: Int J Mol Sci. 2020 May 7;21(9):3303. doi: 10.3390/ijms21093303 (PMC7246652; doi:10.3390/ijms21093303)
Supplement: Supplementary file 1 [file ijms-21-03303-s001.pdf]

Supplementary Figure

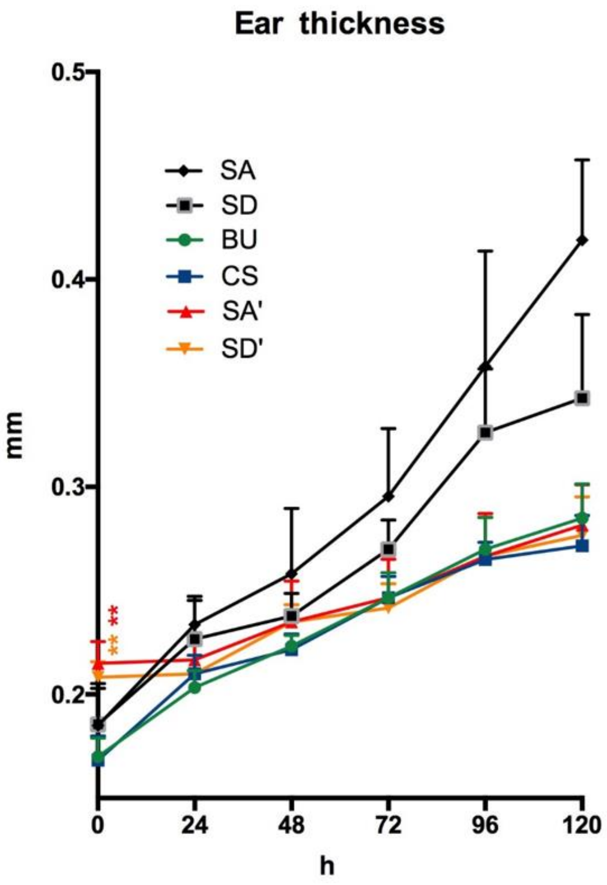


Figure S1: SA′ and SD′ were significantly thicker at 0hrs than BU and CS. At 72 hrs and 120hrs SA′ and SD′ had no significant difference than BU and CS. (** $p < 0.01$)

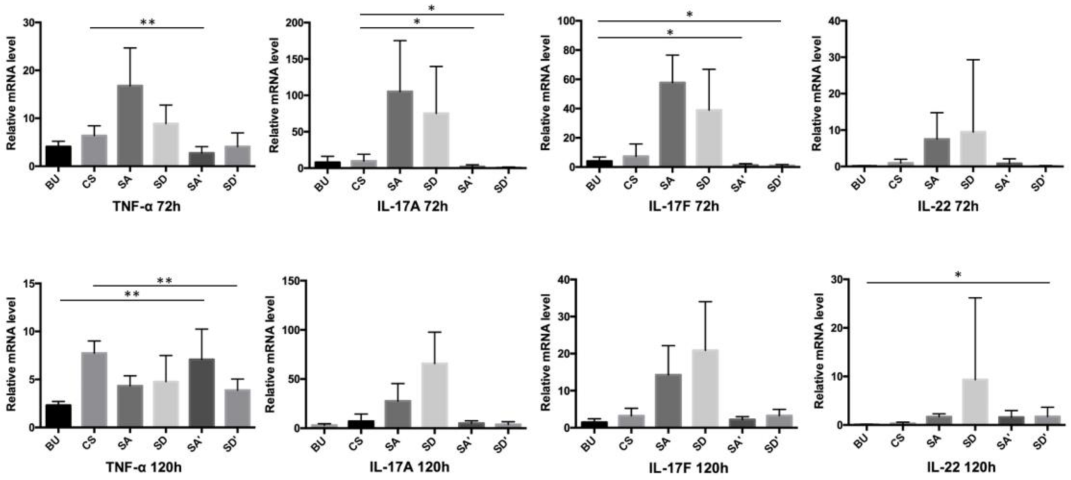

Figure S2: At 72hrs, SA' and SD' presented significantly lower TNF, IL-17A, IL-17F than BU and CS. At 120hrs, TNF- α was higher for SA' than BU but SD' was lower than CS. (* $p < 0.05$, ** $p < 0.01$)

Note: Significance is only compared between SA' and SD' and BU and CS on these figure.
